# Syndrome lymphoprolifératif avec autoimmunité: à propos d’un cas

**DOI:** 10.11604/pamj.2022.43.61.33009

**Published:** 2022-10-07

**Authors:** Houda Ben Youssif, Fatima Ailal, Ibtihal Benhsaien, Jalila El Bakkouri, Laila Jeddane, Khadija El Maani, Ahmed Aziz Bousfiha

**Affiliations:** 1 Laboratoire d´Immunologie Clinique, d´Auto-immunité et d´Inflammation (LICIA), Faculté de Médecine et de Pharmacie, Université Hassan II, Casablanca, Maroc,; 2 Unité d´Immunologie Clinique, Service des Maladies Infectieuses, Hôpital d´Enfants, Centre Hospitalier Universitaire Ibn Rochd, Casablanca, Maroc,; 3 Laboratoire d´Immunologie, Centre Hospitalier Universitaire Ibn Rochd, Casablanca, Maroc,; 4 Service d´hémato-oncologie, Hôpital d´enfants, Centre Hospitalier Universitaire Ibn Rochd, Casablanca, Maroc

**Keywords:** Cytopénies, lymphadénopathie, splénomégalie, cellules T doubles négatives, cas clinique, Cytopenias, lymphadenopathy, splenomegaly, double negative T cells, case report

## Abstract

Le syndrome lymphoprolifératif avec autoimmunité (ALPS) est un trouble hériditaire rare de l´homéostasie lymphocytaire, résultant de mutations dans la voie d´apoptose Fas. Il est caractérisé par une lymphoprolifération chronique non infectieuse et non maligne, et un risque accru de malignité lymphoïde. Le diagnostic de cette pathologie associe généralement une lymphadénopathie et/ou une splénomégalie chroniques qui dépassent 6 mois, des cytopénies autoimmunes, à un taux élevé de lymphocytes Tαβ CD3+CD4-CD8-, appelés lymphocytes T « doubles négatifs ». Le diagnostic différentiel inclut les infections, les maladies auto-immunes ou les tumeurs malignes. Malgré que le tableau clinique et biologique soit fortement évocateur, la maladie semble être méconnue par plusieurs médecins. Nous rapportons pour la première fois un cas d´ALPS au Maroc, âgé de 8 ans avec de la fièvre récurrente, la splénomégalie et des adénopathies. Les examens paracliniques ont révélé une pancytopénie chronique, un excès de lymphocytes TÎ±Î^2^ Double Négative, une hyper gammaglobulinémie, et un taux sérique élevé de Fas ligand soluble. Le diagnostic d´ALPS a été fait. Le traitement de première intention a compris les corticoïdes et les immunoglobulines. Par la suite, la patiente était mise sous mycophénolate, et puis sous sirolimus. Ce dernier a donné une meilleure évolution clinique et biologique. Notre objectif est de sensibiliser les médecins à travers ce rapport de cas à évoquer cette rare pathologie qui est susceptible d´être sous-diagnostiquée.

## Abstract (en)

Autoimmune lymphoproliferative syndrome (ALPS) is a rare genetic disorder of lymphocyte homeostasis, resulting from mutations in the Fas apoptotic pathway. It is characterized by non-infectious and non-malignant chronic lymphoproliferation and an increased risk of lymphoid malignancy. The diagnosis of this condition usually combines chronic lymphadenopathy and/or splenomegaly exceeding 6 months, autoimmune cytopenias, with an elevated level of CD3+CD4-CD8- Tαβ lymphocytes, known as “double-negative” T cells. Differential diagnosis includes infections, autoimmune diseases or malignancies. Although clinical examination and laboratory tests are highly suggestive, this disease goes widely unrecognized. We here report, for the first time, the case of ALPS, a Moroccan patient, and aged 8 years, with recurrent fever, splenomegaly and adenopathies. Paraclinical examinations revealed chronic pancytopenia, higher than normal TÎ±Î^2^ double negative lymphocytes, hypergammaglobulinemia, and elevated serum levels of soluble FAS ligand. The diagnosis of ALPS was made. First-line treatment included corticosteroids and immunoglobulins. Then the patient received mycophenolate followed by Sirolimus. This treatment resulted in better clinical and laboratory tests results. Our aim is to raise awareness of this rare condition, which may be under-diagnosed, among physicians.

## Introduction

Le syndrome lymphoprolifératif avec autoimmunité (*ALPS*) est un trouble de l´homéostasie lymphocytaire associé à des mutations des gènes impliqués aux voies d´apoptose Fas [1]. Il se caractérise par une lymphoprolifération bénigne chronique, des manifestations autoimmunes, et un grand risque de développer un lymphome [2]. Ce syndrome tumoral bénin d´apparition précoce (en moyenne avant cinq ans) associe des adénopathies multifocales, une splénomégalie, et éventuellement une hépatomégalie et il se caractérise par l´accumulation dans le sang et les organes lymphoïdes secondaires d´une population polyclonale de lymphocytes Tαβ matures n´exprimant ni CD4 ni CD8, appelés lymphocytes T « doubles négatifs» (LTαβ DN) [3]. Nous rapportons ici l´observation d´une patiente atteinte d´*ALPS*, âgée de 8 ans, issue d´un mariage consanguin.

## Patient et observation

**Informations relatives à la patiente:** il s´agit d´une fille de 8 ans de parents consanguins (deuxième degré), référée à l´Unité d´Immunologie Clinique (UIC) du Centre Hospitalier Universitaire Ibn Rochd, avec une notion d´allergie en bas âge avec des épisodes de dyspnée sifflante et de prurit cutané récidivant. La patiente n´a aucun cas similaire dans la fratrie. À l´âge de 17 mois, elle commence à faire des fièvres récurrentes survenant par poussée de 4 à 5 jours avec récurrence après 3 semaines, associées à des adénopathies cervicales récidivantes, des aphtes buccaux, et des arthralgies inflammatoires intéressant les mains et les pieds.

**Résultats cliniques:** l´examen clinique lors de la première hospitalisation a retrouvé un nourrisson fébrile avec un développement staturo-pondéral normal. L´examen des aires ganglionnaires a objectivé des adénopathies cervicales bilatérales inflammatoires, mobiles par rapport aux 2 plans, de consistance ferme, indolores d´environ 4 cm. L´examen ORL a montré une gingivo-stomatite avec nécrose des incisives supérieures, aphtes buccaux et une angine érythémato-pultacée. L´examen pleuro-pulmonaire a retrouvé un thorax symétrique, avec présence de râles ronflants et sibilants diffus aux deux champs pulmonaires. Lors de la deuxième hospitalisation, l´évolution était remarquée par la suite par l´apparition à l´âge de 2 ans 10 mois d´une splénomégalie avec un débord splénique de 2 cm, des amas ganglionnaires axillaires bilatéraux, en plus des adénopathies inguinales infra-centimétriques, et des adénopathies cervicales bilatérales de 2 à 3 cm associées à une infiltration de la joue et à une parotidite bilatérale nodulaire.

**Démarche diagnostique:** lors de la première hospitalisation, le bilan inflammatoire était très positif: (la CRP=209 mg/l et la VS= 70 mm la première heure). Le dosage des Immunoglobuline D (IgD) est revenu normal au moment de cet épisode fébrile. À ce stade le diagnostic du syndrome de Marshall a été évoqué devant les signes cliniques et l´amélioration clinique sous les corticoïdes. Lors de la deuxième hospitalisation, l´examen biologique a objectivé l´apparition d´une neutropénie auto-immune et d´une anémie hémolytique auto-immune avec un test de Coombs positif et Anticorps antiPNN positifs. Le myélogramme était riche en hyperplasie de la lignée érythroblastique. Sur le plan radiologique, l´échographie abdominale a montré de multiples ganglions pré-pancréatiques, des hilaires hépatiques et iliaques externes bilatérales de taille infra-centimétriques, le scanner TDM cérébral a révélé une atrophie cortico-sous-corticale, la biopsie de l´adénopathie a montré une hyperplasie lymphoïde réactionnelle. Cette symptomatologie biologique associée à l´apparition de splénomégalie et adénopathies non malignes chroniques a suspecté le diagnostic d´*ALPS*. Les examens paracliniques rentrant dans le cadre du diagnostic d´ALPS ont montré une lymphocytose T CD8 et un excès de lymphocytes TÎ±Î^2^ CD3+CD4-CD8- (LTÎ±Î^2^ Double Négative): 5%, un dosage élevé des immunoglobulines IgA et IgM (IgA: 0,84 g/l (élevé), IgM: 1,01 g/l (élevé), IgG: 4,75 g/l), un taux sérique élevé de Fas ligand soluble (1 ng/ml), et des taux sériques normaux de la vitamine B 12 et de l´interleukine 10 (respectivement: 969 pmol/l et moins de 1 pg/µl). A ce niveau, le diagnostic d´*ALPS* était fait. Nous pourrions même établir le diagnostic d´*ALPS* même en absence de tests de diagnostic moléculaire, ou d´apoptose qui seraient rarement disponibles dans des pays en voie de développement. L´étude génétique était réalisée par la suite, et elle n´a pas permis de mettre en évidence de mutation dans les exons codants du gène Fas et Fas ligand, ainsi que les régions introniques flanquantes. L´étude immuno-histochimique a montré une faible positivité du CD34 (moins de 5%) et un marquage polymorphe PB-PT. L´étude cytogénétique hématologique n´a pas montré d´anomalies. La biopsie ostéo-médullaire (BOM) a mis en évidence une myélodysplasie avec fibrose : les travées osseuses étaient normales avec une moelle riche, les lignées du tissu hématopoïétiques étaient hyperplasiques avec hyperplasie importante de la lignée érythroblastique faite de cellules érythroblastique jeunes sans maturation, la lignée plaquettaire était d´allure dystrophique, la myélofibrose était présente, mais la plasmocytose réactionnelle ou les lésions granulomateuses étaient absentes.

**Intervention thérapeutique et suivi:** la patiente avait des besoins transfusionnels important du fait de l´association d´une anémie centrale et périphérique. Elle était mise initialement sous corticothérapie en pleine dose, et sous perfusion d´immunoglobulines, mais sans amélioration. Ensuite un traitement par le Mycophénolate Mophétil (MMF) a été prescrit pendant 3 mois, ce traitement a été arrêté par la suite devant la persistance des cytopénies. La patiente était donc mise sous rapamycine (sirolimus) à la dose de 1 mg/Jour qui a donné un résultat spectaculaire sur le plan clinique et biologique.

**Perspective du patient:** à l´examen et pendant l´hospitalisation, la patiente avait une humeur dépressive.

**Consentement éclairé:** la maman de la patiente a donné son consentement éclairé.

## Discussion

Depuis sa première description en 1967 par Hale Ören [4], il y a environ 500 cas d´*ALPS* signalés dans le monde chez des personnes de diverses origines raciales et ethniques [5]. L´incidence et la prévalence sont mal connues [6]. De nombreux progrès ont été réalisés dans la compréhension de la physiopathologie, du diagnostic, de la prise en charge et des anomalies génétiques. L´*ALPS* peut être causé par des mutations germinales ou somatiques de Fas, ou des mutations de CASP 10, ou de Fas Ligand [7]. Plus de 70% des patients *ALPS* ont des mutations identifiables dans les gènes de la voie Fas. La plupart de ces patients (62% des patients) ont des mutations germinales de Fas, qui sont généralement transmises par voie autosomique récessive [8].

L´apoptose défectueuse des lymphocytes T entraîne l´expansion des cellules T activées et des cellules T double négatives. Tandis que le défaut de l´apoptose des cellules B entraîne une augmentation de la survie des cellules B productrices d´anticorps conduisant à une hypergammaglobulinémie [9]. Le taux élevé de DNT est considéré comme un grand marqueur de diagnostic pour cette rare pathologie. Notre patiente avait un taux élevé de DNT. Les taux sériques de l´Interleukine10, du Fas ligand soluble et de la vitamine B12 sont généralement élevés chez les patients ALPS présentant des mutations du Fas et peuvent être des biomarqueurs utiles pour ces patients [5,10]. En effet, notre patiente avait des taux normaux de l´IL 10 et de vitamine B12, et elle n´a pas eu de mutation dans le gène Fas.

La lymphoprolifération est la manifestation la plus commune en *ALPS*, elle est illustrée par la lymphadénopathie, la splénomégalie et/ou l´hépathomégalie qui dépassent 6 mois. L´âge moyen de début de ces signes est d´environ 24 mois [11]. Les patients *ALPS* peuvent également avoir des cytopénies multilignées qui sont chroniques et peuvent être réfractaires au traitement. Les patients *ALPS* peuvent présenter initialement des épisodes de fatigue, de pâleur et d´ictère dus à une anémie hémolytique. Ils peuvent également avoir des ecchymoses et des saignements cutanéo-muqueux faciles dus à une thrombocytopénie. Des infections bactériennes peuvent également survenir en raison de la neutropénie [12]. Dans une large cohorte française de 90 patients, la lymphoprolifération était le signe le plus fréquent de la maladie présent chez 89 patients et généralement survenu à un âge précoce. La splénomégalie a été rapportée dans 84% des cas, la lymphadénopathie dans 72%, les cytopénies auto-immunes dans 52% dont l´anémie hémolytique auto-immune était la plus fréquente [12]. La présentation d´enfants présentant une adénopathie généralisée, une splénomégalie et des cytopénies multilignées auto-immunes représente un défi diagnostique car leurs caractéristiques cliniques et biologiques se chevauchent avec celles d´autres troubles hématologiques infantiles. En 1999, des chercheurs des *National Institutes of Health* ont suggéré des critères pour établir le diagnostic d´*ALPS*. La compréhension de la maladie a conduit à la révision des critères de diagnostic et du schéma de classification à la suite du premier atelier international *ALPS* tenu aux *National Institutes of Health* en 2009 (Tableau 1) [13].

**Tableau 1:** critères diagnostiques révisés pour le diagnostic du syndrome lymphoprolifératif avec autoimmunité, basé sur le premier workshop international en 2009 [5]

**principaux**		Lymphadénopathie et/ou splénomégalie chroniques (˃6 mois)
		Taux élevé de TCRαβ, CD3+, CD4-, CD8- (˃ 1,5% du total des lymphocytes ou 2,5% des lymphocytes CD3+)
**accessoires**	primaires	Apoptose lymphocytaire anormale
		Mutation somatique ou germinale de FAS, FASLG, ou CASP10
	secondaires	Taux plasmatique élevé de: FAS Ligand soluble ou Interleukine 10 ou un taux plasmatique ou sérique élevé de V B12 (respectivement ˃200 pg/mL, ˃20 pg/mL, ˃1500 pg/mL)
		Résultats immunohistologiques typiques
		Cytopénie autoimmune (anémie hémolytique, thrombocytopénie ou neutropénie) et un taux élevé des Immunoglobulines
		Antécédents familiaux d´une lymphoprolifération non maligne, non infectieuse avec ou sans autoimmunité

Un diagnostic définitif de l´ALPS repose sur la présence des deux critères principaux, et un critère accessoire primaire. Un diagnostic probable est basé sur la présence d´un critère principal et un critère accessoire secondaire [5]

La prise en charge des patients *ALPS* vise à contrôler les cytopénies auto-immunes. Le traitement de première intention de l´*ALPS* comprend souvent des injections intraveineuses de corticoïdes et d´immunoglobulines (IVIgG). Les plus couramment utilisés dans l´*ALPS* sont le mycophénolate mofétil (MMF) et le sirolimus. Le MMF est le plus indiqué pour traiter les manifestations auto-immunes telles que les cytopénies [5]. Le sirolimus s´est avéré efficace dans le traitement des cytopénies réfractaires, ainsi que d´autres manifestations auto-immunes de l´*ALPS* chez les patients ayant mal répondu aux corticoïdes et (IV Ig) [14]. La morbidité et la mortalité dans l´*ALPS* dépendent de la sévérité de l´auto-immunité, ou de l´hypersplénisme associé [1^5^].

## Conclusion

Enfin, Ce cas souligne à nouveau le fait que les pédiatres doivent garder la possibilité d´un diagnostic rare tel que l´*ALPS*. L´*ALPS* doit être pris en compte dans le diagnostic différentiel des nourrissons et des enfants présentant une lymphadénopathie, une splénomégalie et une cytopénie autrement inexpliquée. La suspicion doit être renforcée si la cytopénie est à médiation immunitaire. Une plus grande sensibilisation à l´*ALPS* devrait se traduire par une approche diagnostique plus ciblée et une prise en charge plus précoce de cette pathologie.
